# Supplementary material for: Can the Addition of Maintenance Electroconvulsive Therapy to Pharmacotherapy Improve Relapse Prevention in Severe Major Depressive Disorder? A Randomized Controlled Trial
Source: Brain Sci. 2021 Oct 11;11(10):1340. doi: 10.3390/brainsci11101340 (PMC8534103; doi:10.3390/brainsci11101340)

Data supplement.

Figure S1. Kaplan-Meier function of the cumulative probability of remaining in remission at 15 months (log-rank test = 0.143).

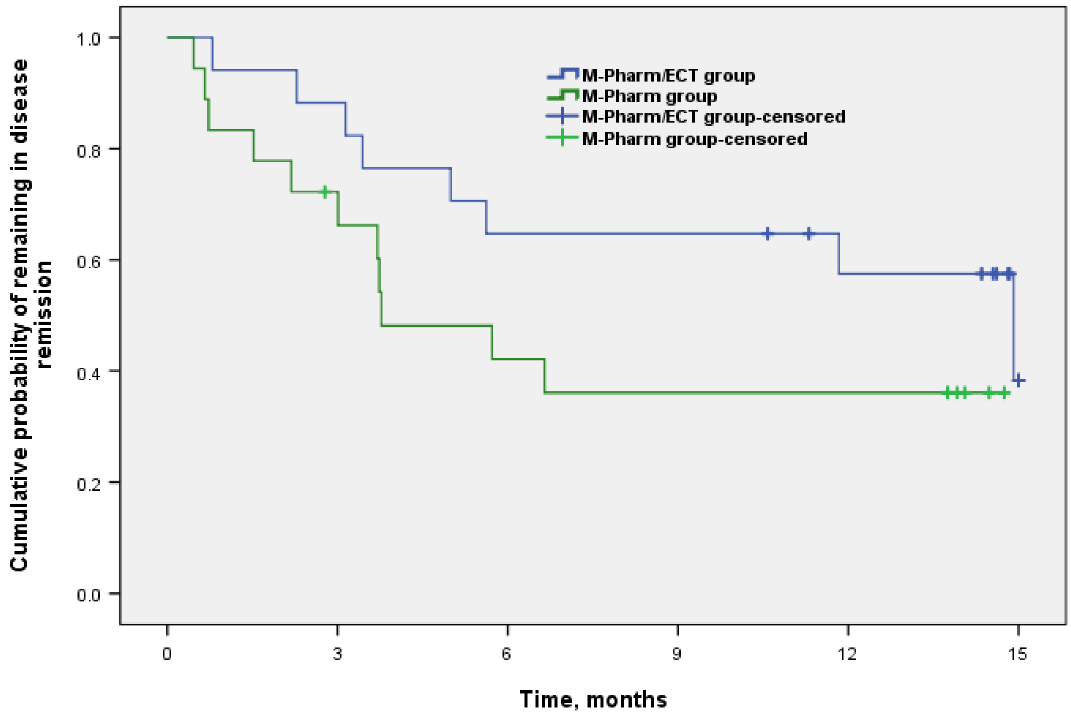

Supplement: Supplementary file 1 [file brainsci-11-01340-s001.zip › brainsci-1414989-suppl.pdf]
